# Effects of ketogenic diet on the clinical and electroencephalographic features of children with drug therapy-resistant epilepsy

**DOI:** 10.3892/etm.2012.823

**Published:** 2012-11-22

**Authors:** BAOMIN LI, LILI TONG, GUIJUAN JIA, RUOPENG SUN

**Affiliations:** Department of Pediatrics, Qilu Hospital of Shandong University, Jinan, Shandong 250012, P.R. China

**Keywords:** epilepsy, refractory, ketogenic diet, efficacy, electroencephalogram

## Abstract

The aim of this study was to investigate the effects of a ketogenic diet (KD) on the clinical and electroencephalographic (EEG) features of children with drug therapy-resistant epilepsy. A total of 31 children with drug therapy-resistant epilepsy were selected, including 19 males and 12 females. The youngest was 7 months old and the oldest was 7 years old. Clinical seizures in the children prior to and 1 week, 1 month and 3 months after the initiation of the KD were compared and the clinical effect of the KD was evaluated. The ratio of fat to carbohydrate + protein in the KD was 4:1. Following the initiation of the KD treatment, the original antiepileptic drugs were not changed. The changes in occipital region background rhythm and interictal spike wave discharge index (SI) were evaluated prior to and 1 week, 1 month and 3 months after the initiation of the KD. The efficacy had an upward trend over time, with a total efficacy rate of 51.61% 1 week later, 67.74% 1 month later and 70.97% 3 months later. Doose syndrome showed the greatest response to KD, with a 100% efficacy rate. However, since there were only two cases in the study, its efficacy remains to be assessed. Infantile spasm also showed a good response to KD; 9 of the 16 patients were seizure free and the total efficacy rate was 81.25%. As the length of the KD treatment was increased, the background rhythms of the children underwent significant changes and the SI was significantly reduced. KD not only demonstrated good clinical efficacy, but also significantly reduced the frequency of interictal epileptic discharges and improved the EEG background rhythm.

## Introduction

Drug-resistant childhood epilepsy accounts for 20–30% of cases of epilepsy. Although a growing number of new antiepileptic drugs have been developed, the incidence of drug-resistant childhood epilepsy has not been reduced significantly (1,2). In addition to antiepileptic drugs, treatments for drug-resistant childhood epilepsy include ketogenic diet (KD), surgery and vagus nerve stimulation therapy. The KD is a diet program with a high ratio of fat, low carbohydrate content and adequate protein so that the body mainly depends on fat rather than carbohydrates to supply energy. Brain diseases may be treated by starvation using catabolic energy (mainly fat) *in vivo*. Use of the KD method for the treatment of refractory epilepsy was first reported in 1921. Since then, the results of a number of clinical and animal studies have demonstrated that KD treatment has good efficacy. At present, an increasing number of epilepsy studies are focusing on KD treatment due to its low cost, small risk and good efficacy.

The mechanism underlying the treatment of epilepsy using the KD is not clear (3,4). The results of our early research suggested that the antiepileptic mechanism of KD may be related to an increase of GluR5 expression in the CA1 region of the intermediate neurons of young rats, leading to the enhancement of inhibitory synaptic transmissions in the hippocampus, thus preventing the propagation of seizures (5). Bough *et al*(6) demonstrated that the efficacy of the KD is age-dependent, that is, the younger the child, the more marked the antiepileptic effect. Thus, the KD treatment for epilepsy is mainly used in pediatrics.

Previous studies have revealed that KD has a good efficacy for multiple seizure types (7–11). In at least half of patients treated with KD, the seizure frequency has been reduced by >50%. KD not only effectively alleviates clinical seizures in patients with epilepsy, but also improves electroencephalographic (EEG) features (9–10). Since November 2009, the treatment of intractable epilepsy in children using the KD method has been performed in 48 children with refractory epilepsy at the Qilu Hospital of Shandong University. In the present study, the clinical data and EEG information of 31 participants who adhered to the treatment for 3 months or more are reported.

## Patients and methods

### Clinical data

A total of 31 cases diagnosed as refractory epilepsy were selected for the present study. The patients had received two or more types of regular antiepileptic drugs, but frequent seizures continued (>4 times/week). The present study was conducted in accordance with the Declaration of Helsinki and with the approval of the Ethics Committee of Qilu Hospital of Shandong University. Written informed consent was obtained from the legal guardians of all participants. The participants included 19 males and 12 females. The youngest was 7 months old, the oldest was 7 years old, and the average age was 2 years and 5 months. Among the participants, there were: 16 cases of infantile spasm, of which one had a family history of epilepsy, four had a history of neonatal hypoxic-ischemic encephalopathy and two had tuberous sclerosis; 11 cases of Lennox-Gastaut syndrome (LGS), in which two had a family history of epilepsy, one had a family history of febrile convulsions, one had a history of traumatic brain injury and two had viral encephalitis sequelae; 2 cases of Doose syndrome; and 2 cases of Dravet syndrome, one of which had a family history of febrile convulsions. The psychomotor development of 26 cases lagged behind that of healthy children of the same age while the remaining 5 cases had normal psychomotor development. All patients underwent imaging examination and abnormalities were observed in 9 cases, of which two had tuberous sclerosis, two had ventricular dilatation, one had Dandy-Walker malformation and four had cerebral hypoplasia. The parents of the patients ensured adherence to the KD.

### Recording of clinical seizures and medication use prior to the KD treatment

The seizure frequency and the antiepileptic drugs used one month prior to the KD treatment were recorded in detail.

### KD treatment

When the patients were admitted to the hospital, auxiliary examinations were completed to exclude fat or ketone body metabolism disorders or mitochondrial diseases, as well as severe heart, lung or blood diseases, liver function damage, hyperlipidemia and urinary stones. Fasting was performed for 24–48 h during which the vital signs, trace amounts of blood glucose and urine ketone were monitored. Orange juice was provided if the blood glucose level was <2.1 mmol/l. The KD treatment was administered when the urinary ketone level reached ≥3 mmol/l. Ketogenic milk was provided for children ≤3-years-old. A KD schedule according to the KD software (KD catering software, www.szbos.net.cn) was used for children >3-years-old and the ratio of energy produced by fat to carbohydrate and protein was 4:1. The daily calorie and protein requirements were supplied and the full amount of the KD was administered from the start of the study. Calories (251.2–334.9 kJ/kg) and protein (1–2 g/kg) were divided into three portions and consumed three times a day. During the KD treatment, the vital signs and trace amounts of blood glucose, blood ketone and urine ketone were closely monitored. The form and frequency of seizures and adverse reactions were observed and recorded. Routine blood tests, liver function and blood lipids were regularly reviewed. The children's parents were trained to prepare the KD food or, in certain cases, were allowed to create their own recipes using the KD software.

### Antiepileptic drug use

Following the initiation of the KD, the antiepileptic drugs and doses used were not changed from those administered previously.

### Efficacy evaluation of the KD treatment

The seizure frequencies of the children prior to and 1 and 3 months after the KD treatment were analyzed. The efficacy was evaluated as completely seizure free, markedly effective (seizure reduction ≥75%), effective (seizure reduction ≥50%) or invalid (seizure reduction <50%). The total efficacy rate = (number of completely seizure free + markedly effective + effective cases) ÷ total number of cases x 100.

### Evaluation of the effect of KD on EEG features

Prior to and 1 week, 1 month and 3 months after the KD treatment, ≥12 h of video-EEG (VEEG) monitoring results were examined. The background rhythm in the occipital region under the awake and quiet states and changes of interictal spike wave index (SI) were evaluated.

### SI was an average of spike discharge times within 1 sec

In the calculation method used, 100 sec of the EEG results under the awake and quiet states (without artifact fragments) were selected and the number of spikes (n) contained in the EEG recording was calculated. SI=n/100.

## Results

### Clinical seizures and medication use prior to KD treatment

The seizure frequency ranged between once every 2–3 days and >20 times/day. The 31 participants underwent regular treatment using two or more types of antiepileptic drugs and the efficacy was not evident (Table I).

### Efficacy of KD

The efficacy of the KD treatment had an upward trend over time. The total efficacy rate 1 week after the KD treatment was 51.61% (seizure free, 9 cases; markedly effective, 4 cases; effective, 3 cases; and invalid, 15 cases). The total efficacy rate was 67.74% at 1 month (seizure free, 15 cases; markedly effective, 5 cases; effective, 1 case; and invalid, 10 cases). The total efficacy rate was 70.97% at 3 months (seizure free, 14 cases; markedly effective, 5 cases; effective, 3 cases; and invalid, 9 cases; Table II).

### Epilepsy syndrome and efficacy 3 months after treatment

The results showed that different epilepsy syndromes responded differently to the KD therapy. The greatest response to the KD therapy was for Doose syndrome which had an efficacy rate of 100%. As there were few cases of the syndrome in this study, its efficacy remains to be assessed. Furthermore, infantile spasms responsed well to the KD treatment; 9 or the 16 cases were seizure free and the total efficacy rate was 81.25%.

For LGS, the total efficacy rate was 54.55% (4 of 11 cases were seizure free). There were 2 cases of Dravet syndrome KD was markedly effective in one case and invalid in the other. The details for the different epilepsy syndromes are shown in Table III.

### Changes of EEG features following the KD treatment

As the length of the KD treatment was increased, significant changes in the background rhythms of the children were observed. The four children with infantile spasms had highly imperfect rhythms prior to the KD treatment but normal background rhythms were detected 1 month after the KD treatment. The SI was significantly reduced. The interictal spike firing frequency was also significantly reduced. At 1 week, a reduction in the SI of >50% was observed in 5 of the 31 cases; this increased to 14 cases at 1 month and 16 cases at 3 months. The details are shown in Tables IV and V.

## Discussion

Refractory childhood epilepsy refers to seizures that are not completely controlled by treatment with two or more types of regular antiepileptic drugs and is also known as drug-resistant childhood epilepsy. The KD is an effective treatment for drug-resistant epilepsy in addition to treatments such as surgery and vagus nerve stimulation therapy. The KD treatment has a good efficacy on a variety of seizure types. Beniczky *et al*(11) reported that in the application of KD in the treatment of 50 cases of intractable epilepsy, the seizure frequency in 66% of the patients (33/50) was reduced by >50% and in 36% (18/50) was reduced by >90%, while 9 cases were seizure free. In the study by Hallböök *et al*(9), 18 patients were treated with the KD. The results showed that clinical seizures in 44% of the patients were reduced by >90% and 22% of the patients were completely seizure free. In the present study, the KD treatment of 31 cases of drug-resistant childhood epilepsy was observed. The results showed that clinical seizures in >70% the children were reduced by >50% and 14 cases were completely seizure free following 3 months of treatment, which was consistent with the results of previous studies. Kossoff *et al*(12) considered that KD had particular efficacy for epilepsy syndromes such as myoclonic-astatic epilepsy (MAE), Dravet syndrome, Rett syndrome and West syndrome (particularly combined with tuberous sclerosis). A number of studies (13–16) also revealed that KD was more effective for Dravet and Doose syndromes and resulted in a higher ratio of seizure-free patients. The data in the present study indicate that Doose syndrome had the greatest response to the KD treatment. However, as there were few cases of this syndrome in the present study, its efficacy remains to be assessed. Infantile spasm also responsed well to the KD treatment; of the 16 cases treated, nine were seizure free and the clinical seizures in >80% of the patients (13/16) were reduced by >50%. Hallböök *et al*(9) observed that the efficacy of KD for partial seizures was poor. Out of 18 cases, 7 withdrew after 3 months of treatment, 4 due to poor efficacy and 3 due to adverse reactions. Beniczky *et al*(11) demonstrated that the efficacy of KD for complex partial seizures of temporal lobe epileptic discharge was poor. In the current study, three children with refractory partial epilepsy underwent KD treatment. No significant improvements in their seizures were observed so they withdrew from treatment. The results suggested that KD should be selected for the appropriate seizure types in order to achieve greater effects.

KD not only effectively alleviates the clinical seizures of epilepsy patients, but also markedly improves the EEG features of epilepsy patients. Hallböök *et al*(9) observed that the interictal epileptic discharge frequency decreased significantly following 3 months of KD treatment. Remahl *et al*(10) compared 24 h EEG data prior to and following KD treatment in 23 cases and 65.2% (15/23) of patients experienced a significant reduction of epileptic interictal discharge frequency with normal background rhythms. Changes in EEG features may occur in all patients and the EEG change may be evident even in patients with a poor clinical KD efficacy. Dressler *et al*(17) also reported that 6 months after KD treatment, the patients' EEG interictal epileptiform discharge frequencies and background rhythms were improved significantly. Kessler *et al*(18) studied 48 cases of KD treatment and observed that in the majority of patients' EEG recordings, the sober period epileptiform discharges after 1 month of treatment were reduced significantly and continued to reduce after 3 months, but this EEG improvement was more evident in patients with a good KD efficacy. The present data also shows that as the length of KD treatment was increased, the background rhythm of the children improved significantly. In the 4 cases of infantile spasms, the background rhythms were highly imperfect prior to KD therapy, but 1 month later the background rhythms were detected and 3 months later the rhythms were more normal. A reduction in the SI of >50% was observed in >50% (16/31) of the patients. Researchers (18–20) have suggested that this EEG change results from favorable electrical physiological effects of the KD, similar to the effect of antiepileptic drugs on the brain, and that this existed in the patients' EEG physiology even if the efficacy of the KD treatment was poor. This indicated that the KD had a positive impact on the central nervous system and cortical neurons, suggesting that the KD may be used for the treatment of certain epileptic encephalopathy, such as epilepsy with continuous spikes and wave during slow sleep (CSWS) and Landau-Kleffner syndrome (LKS), in order to improve the patient's brain function.

In summary, the KD is important in the treatment of patients with refractory epilepsy, particularly in children. KD not only has good clinical efficacy, but also significantly reduces the frequency of interictal epileptiform discharges and improves the EEG background rhythm, thereby simultaneously reducing clinical seizures and improving the brain function and quality of life of the patients.

## Figures and Tables

**Table I.** Patient clinical seizures and drug treatment within 1 month prior to ketogenic diet (KD) treatment.

	Seizure frequency (times/day)				Antiepileptic drugs		
	0.3–5	5–10	10–20	≥20	2 types	3 types	≥4 types
Cases	6	5	11	9	10	12	11

**Table II.** Efficacy of the ketogenic diet (KD).

	Efficacy [n (%)]				Total efficacy [n (%)]	
Diet duration	Seizure free	Markedly effective	Effective	Invalid	Effective	Invalid
1 week	9 (29.03)	4 (12.90)	3 (9.68)	15 (48.39)	16 (51.61)	15 (48.39)
1 month	15 (48.39)	5 (16.13)	1 (3.23)	10 (32.26)	21 (67.74)	10 (32.26)
3 months	14 (45.16)	5 (16.13)	3 (9.68)	9 (29.03)	22 (70.97)	9 (29.03)

**Table III.** Epilepsy syndrome and efficacy.

		Efficacy [n(%)]				Total efficacy [n(%)]	
Syndrome	n	Seizure free	Markedly effective	Effective	Invalid	Effective	Invalid
Infantile spasms	16	9 (56.25)	3 (18.75)	1 (6.25)	3 (18.75)	13 (81.25)	3 (9.68)
LGS	11	4 (36.36)	1 (9.09)	1 (9.09)	5 (45.45)	6 (54.55)	5 (45.45)
Doose Syndrome	2	1 (50.00)	0 (0)	1 (50.00)	0 (0)	2 (100)	0 (0)
Dravet Syndrome	2	0 (0)	1 (50.00)	0 (0)	1 (50.00)	1 (50)	1 (50)
Total	31	14 (45.16)	5 (16.13)	3 (9.69)	9 (29.03)	22 (70.97)	9 (29.03)

LGS, Lennox-Gastaut syndrome.

**Table IV.** Changes of background rhythm frequency [n (%)].

Diet duration (n)	Plus ≥2 Hz	Plus 1–2 Hz	No change (<1 Hz)
1 week (31)	0 (0)	3 (9.68)	28 (90.32)
1 month (31)	3 (9.68)	7 (22.58)	21 (67.74)
3 months (31)	9 (29.03)	15 (48.39)	7 (22.58)

**Table V.** Changes of SI [n (%)].

Diet duration (n)	Reduction ≥75%	Reduction 75–50%	Reduction 50–30%	Reduction <30%
1 week (31)	1 (3.23)	4 (12.90)	12 (38.71)	14 (45.16)
1 month (31)	8 (25.81)	6 (19.35)	4 (12.90)	13 (41.94)
3 months (31)	14 (45.16)	2 (6.45)	3 (9.68)	10 (32.26)

SI, interictal spike wave index.
